# A case of angle‐closure glaucoma caused by spontaneous lens dislocation

**DOI:** 10.1002/ccr3.6670

**Published:** 2022-12-05

**Authors:** Keisuke Kondo, Hiroki Isono

**Affiliations:** ^1^ Department of General Medicine HITO Medical Center Ehime Japan; ^2^ Department of General Medicine Institute of Biomedical Science, the University of Tokushima Graduate school Tokushima Japan

**Keywords:** angle‐closure glaucoma, loss of vision, spontaneous lens dislocation, sudden headache

## Abstract

Spontaneous lens dislocation without genetic factors is rare. In this case, spontaneous lens dislocation occurred without an apparent trigger and resulted in secondary angle‐closure glaucoma. A head computed tomography (CT) scan showed lens dislocation. It is critical to assess for lens dislocation during head CT scan for a sudden headache.

## Abstract 2

In this case, spontaneous lens dislocation occurred without an apparent trigger. A CT scan of the head showed that the left intraocular lens had deviated posteriorly.

## 1 CASE

**FIGURE 1:**
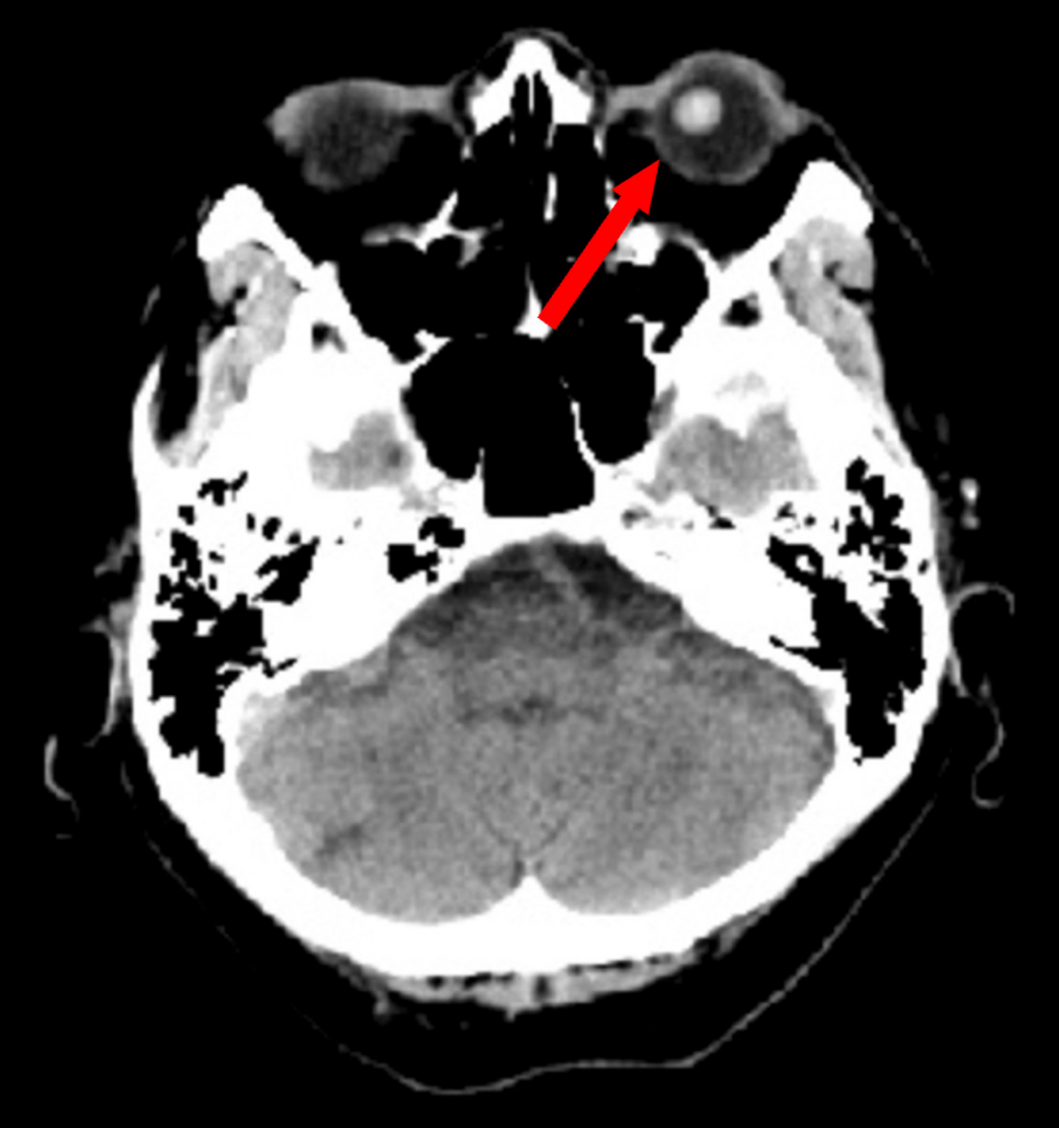
Left intraocular lens is deviated to the left posteriorly.

A healthy man in his 70 s, who suddenly lost vision in his left eye while lying down and watching TV, presented to the emergency room of a general hospital. He also experienced intense pain radiating from the left eye to the left side of the head. Examination of the left eye could not be done because he was unable to open the eye due to pain. The sudden onset headache and loss of vision suggested, in addition to glaucoma attack, a possibility of an intracranial disease as differential. A computed tomography (CT) scan of the head was performed to rule out subarachnoid hemorrhage. It showed that the left intraocular lens had deviated posteriorly (Figure 1). The patient was immediately transferred to the ophthalmology department where he was diagnosed with an acute glaucoma attack caused by lens dislocation and underwent left vitrectomy.

## 2 DISCUSSION

In this case, spontaneous lens dislocation occurred without an apparent trigger; obstruction of aqueous humor pathway resulted in secondary angle‐closure glaucoma. The commonest cause of lens dislocation is trauma. Spontaneous lens dislocation without predisposing genetic factors is rare.^1^, ^2^ Head CT may be useful for diagnosing lens dislocation in patients with a sudden loss of vision accompanied by ocular pain.

## AUTHOR CONTRIBUTIONS

Dr Keisuke Kondo and Dr Hiroki Isono prepared, organized, wrote, and edited all aspects of the manuscript. Dr Keisuke Kondo prepared all of the histology figures in the manuscript. All authors contributed equally toward preparing the manuscript and participated in the final approval of the manuscript before its submission.

## FUNDING INFORMATION

The authors did not receive funding for this article.

## CONFLICT OF INTEREST

None declared.

## ETHICAL APPROVAL

All procedures performed were in accordance with the ethical standards. The examination was made in accordance with the approved principles.

## CONSENT

This article is published with the written consent of the patient.

## Data Availability

None.
